# A scoping review and thematic analysis of the effects of tropical cyclones on diarrheal diseases

**DOI:** 10.1097/EE9.0000000000000366

**Published:** 2025-01-09

**Authors:** Szu Yu Lin, Paul L. C. Chua, Lei Yuan, Nasif Hossain, Jinyu He, Lisa Yamasaki, Lina Madaniyazi, Chris Fook Sheng Ng, Aurelio Tobias, Masahiro Hashizume

**Affiliations:** a Department of Global Health Policy, Graduate School of Medicine, The University of Tokyo, Tokyo, Japan; b Center for Climate Change Adaptation, National Institute for Environmental Studies, Tsukuba, Japan; c Center Hospital of the National Center for Global Health and Medicine, Tokyo, Japan; d School of Tropical Medicine and Global Health, Nagasaki University, Nagasaki, Japan; e Institute of Environmental Assessment and Water Research (IDAEA), Spanish Council for Scientific Research (CSIS), Barcelona, Spain

**Keywords:** Tropical cyclone, Extreme weather event, Diarrheal disease, Infectious disease, Environmental epidemiology, Scoping review, Thematic analysis

## Abstract

**Background:**

Tropical cyclones pose significant health risks and can trigger outbreaks of diarrheal diseases in affected populations. Although the effects of individual hazards, such as rainfall and flooding, on diarrheal diseases are well-documented, the complex multihazard nature of tropical cyclones is less thoroughly explored. To date, no dedicated review comprehensively examines the current evidence and research on the association between tropical cyclones and diarrheal diseases.

**Methods:**

We performed a scoping review to map the literature on tropical cyclones and diarrheal diseases. A comprehensive literature search was performed across multiple online databases, including PubMed/MEDLINE, Web of Science, Scopus, Google Scholar, and ProQuest. We then performed a thematic analysis on the specific transmission pathways between tropical cyclones and diarrheal diseases as described in the literature.

**Results:**

A total of 96 studies were included and categorized in this scoping review. Of these, 23 studies quantitatively assessed the association between tropical cyclones and diarrheal diseases, with more than half reporting a positive association. Additionally, we identified 30 studies that detailed transmission pathways, which we used for thematic analysis. Significant variability was observed in the definition of tropical cyclone exposure, with studies using different criteria such as an event, wind speed, or rainfall. Most studies used pre-post comparison designs without concurrent control groups, which can introduce limitations affecting internal validity by not accounting for temporal confounders. Diarrheal diseases can either increase or decrease during and after tropical cyclones, depending on the specific pathogens and the different strengths of tropical cyclones.

**Conclusion:**

The variability in exposure definitions and study designs impedes the ability to quantitatively pool evidence. To improve the comparability and reliability of future research, we recommend that studies explore how different tropical cyclone exposure definitions impact results to identify the most appropriate metrics. We also suggest adopting more robust study designs, such as difference-in-difference or controlled interrupted time series for studying single tropical cyclone events, and case-crossover designs for studying multiple events. Additionally, studies examining specific causal pathways, such as integrating environmental sampling with health outcomes, should be explored to identify effective prevention strategies.

**What this study adds**

This study enhances our understanding of the impact of tropical cyclones on diarrheal diseases by analyzing existing literature. We identified critical inconsistencies, such as varying definitions of tropical cyclone exposure and diverse study designs. The lack of standardized definitions and simplified methods that overlook time-varying confounders can lead to biased results. We advocate for a unified definition and more sophisticated study designs to improve the understanding of the multihazard effects of tropical cyclones on diarrheal diseases. Moreover, specific transmission pathways between tropical cyclones and diarrheal diseases remain inadequately understood.

## Introduction

Tropical cyclones are low-pressure systems that develop over tropical waters.^1^ These powerful storms pose multiple hazards, including strong wind, heavy rainfall, storm surge, and flooding, leading to numerous casualties, deaths, and significant damage to property and infrastructure each year. Globally, over the past 50 years, tropical cyclones have been responsible for 1945 disasters, claiming 779,324 lives and causing US$ 1.4 trillion in economic losses.^2^ During this period, tropical cyclones accounted for 17% of all weather-, climate-, and water-related disasters, underscoring their significant impact on human life and the economy.^2^ Previous studies have examined the effects of tropical cyclones on a range of health outcomes, including all-cause mortality,^3–9^ preterm births,^10^ cardiovascular diseases,^11,12^ respiratory health diseases,^11–13^ kidney diseases,^14^ and infectious diseases.^15,16^

Among these health outcomes, diarrheal diseases stand out as particularly important in relation to tropical cyclones. Diarrheal diseases, as defined by the World Health Organization, are characterized by an individual passing three or more loose or liquid stools in a day, typically resulting from infections in the intestinal tract caused by various bacteria, viruses, and parasites.^17^ Diarrheal diseases are often spread through contaminated water or from person to person due to poor hygiene. Hence, tropical cyclones indirectly impact them through major disruption of water systems and sanitation infrastructure. Given that diarrheal diseases are a serious public health concern – ranking as the 14th leading cause of death in 2021 – it is crucial to understand how they interact with tropical cyclones.^18^

The relationship between diarrheal diseases and rain-related environmental factors has been thoroughly studied in a meta-analysis.^19^ Both excessively wet and dry conditions can heighten the risk of diarrheal diseases by altering pathogen concentrations and water sources. Notably, extreme rainfall following a dry period can flush accumulated pathogens into water sources, increasing disease spread.^19^ Pathogens respond differently to rainfall: bacterial and parasitic infections are generally more common during rainy seasons, as these conditions support pathogen survival in water. However, the impact of rainfall on viral infections varies by pathogen. For instance, norovirus hospitalization rate increased with rainfall,^20^ while rotavirus incidence^19^ and hospitalization risk^20^ decreased.

Tropical cyclones are multihazard extreme events making them more complex in nature and challenging to study. To our knowledge, there is no review study specifically dedicated to the relationship between tropical cyclones and diarrheal diseases, but there are five review studies that touch on the topic.^19,21–24^ While these studies offer valuable insights into the broader interactions between climate factors and waterborne diseases, they generally focus on the impacts of heavy rainfall and flooding, with only incidental mentions of tropical cyclones. Given the intricate relationship between diarrheal diseases and tropical cyclones, a dedicated study specifically addressing this relationship is warranted.

This scoping review aims to comprehensively analyze the available literature on the association between tropical cyclones and diarrheal diseases. The primary objective is to examine various methodological approaches employed in quantifying this association. The second objective is to elucidate the pathways through which exposure to tropical cyclones contributes to the incidence of diarrheal diseases by utilizing a thematic analysis. By identifying gaps in current research on this topic, our goal is to offer valuable insights for guiding future research.

## Materials and methods

This scoping review followed the Joanna Briggs Institute guidelines.^25^ It was conducted following a protocol^26^ in accordance with the Preferred Reporting Items for Systematic Reviews and Meta-Analyses for Scoping Review (PRISMA-ScR) reporting guidelines.^27^

### Search strategy

We used online databases of PubMed/MEDLINE, Web of Science, Scopus, Google Scholar, and ProQuest to perform the literature search on 23 August 2023. The search terms used refer to tropical cyclones (e.g., tropical cyclone, tropical depression, tropical storm, hurricane, and typhoon) and diarrheal diseases (e.g., diarrhea, enteric infection, and gastroenteritis). The detailed search query is provided in the Supplementary Text 1; http://links.lww.com/EE/A321.

### Eligibility criteria

We included all types of published research, without limiting the search to a specific time period. Review studies, such as narrative, scoping, and systematic reviews, were also included. Gray literature, such as reports, conference abstracts, and editorials, was included. Only articles written in English were included. We excluded presentation slides, newspaper articles, and research articles without accessible full text.

#### Participants

We included all human-related studies irrespective of age, sex, health status, and geographic location. Studies on animals, plants, and pathogens without the involvement of humans were excluded.

#### Exposure

We encompass all tropical cyclones occurring in all tropical cyclone basins.^22^ We excluded extra-tropical cyclones (i.e., mid-latitude cyclones that occur outside the tropical locations), monsoons, and tornadoes. We excluded floodings that are not caused by tropical cyclones.

#### Outcome

We included all kinds of diarrheal disease-related health outcomes, including mortality, hospital admissions, healthcare facility visits, ambulance transport, emergency room visits, and clinically diagnosed or self-reported symptoms cases. Also, we included pathogen-specific diarrheal diseases listed in the 10th revision of the International Statistical Classification of Diseases (ICD-10) A00 to A09.

#### Context

All articles that directly described the association between tropical cyclones and diarrheal diseases were considered in this scoping review.

### Study selection, data extraction, and summary of results

All records from the literature search were imported and deduplicated using EndNote 20 (Clarivate, Philadelphia, PA). At least two reviewers (S.Y.L., L.Y., N.H., and J.H.) independently screened the titles and abstracts based on the inclusion and exclusion criteria before full-text review. Two reviewers (S.Y.L. and P.L.C.C.) independently performed a full-text review. Any disagreements between the evaluations made by the reviewers were resolved through discussion or feedback from a third reviewer (M.H.). Rayyan (Cambridge, MA), a web and mobile app for systematic and scoping reviews, was used to perform the screening process.^28^ A PRISMA flow diagram was created to show the inclusion process. A data extraction form was created within Notion (Notion Labs Inc., San Francisco, CA) (a productivity software) and reviewers (S.Y.L. and L.Y.) extracted the following data for each included study:

(1) Citation details (e.g., first author and affiliation, title, journal, and publication date)
(2) Study area and period
(3) Characteristics of the study population (e.g., children, elderly, or evacuees)
(4) Tropical cyclone definition and data source(s)
(5) Diarrheal diseases definition, type of health outcome (e.g., surveillance, hospital admissions, or healthcare facility visits), and data source(s)
(6) Study design and statistical analysis
(7) Direction of association (i.e., positive, negative, or mixed)
(8) Statement of presumed mechanism(s) associating tropical cyclones with diarrheal diseases

We used the World Bank regions for the categorization of the study area.

After categorizing all studies, we conducted a further analysis of the epidemiological studies, with a primary focus on reviewing their methods and results.

### Thematic analysis of transmission pathways

The objective of this thematic analysis is to elucidate the transmission pathways linking tropical cyclones to diarrheal diseases.^24,29^ For this purpose, qualitative data were drawn from 30 of the 96 articles that specifically discussed these transmission pathways (see Supplementary Table 4; http://links.lww.com/EE/A321 for the list of articles).

We opted for an iterative-inductive thematic analysis, starting with a bottom-up coding approach to allow themes to emerge naturally from the data rather than being predefined.^30^ Our process began with a thorough review of each included article to gain a comprehensive understanding of their content and extract relevant information on transmission pathways. Initial codes were generated using an in vivo process, with code names closely reflecting the original language used in the articles, preserving the authenticity of the data.

Following open coding, we applied axial coding (referred to as subthemes in Supplementary Table 4; http://links.lww.com/EE/A321), to organize open codes into broader categories.^30^ We assigned descriptive codes to capture various perspectives, such as human behavior (e.g., unsafe water handling practices), pathogen dispersion, and environmental factors (e.g., damage to water systems). From these axial codes, we identified the highest-level codes that represent direct exposures caused by tropical cyclones.^30^ These were recognized as the main themes, as outlined in Supplementary Table 4; http://links.lww.com/EE/A321.

### Quality assessment

In line with scoping review guidelines, study quality or formal risk of bias was not assessed nor used as a basis for study exclusion.^27,31^

### Ethical approval

We did not apply for ethical approval because this scoping review was confined to a descriptive narrative analysis of publicly available studies and gray literature. This scoping review protocol is registered in the Open Science Framework at https://osf.io/qmnft/.

## Results

### Review of studies

**Figure 1.**
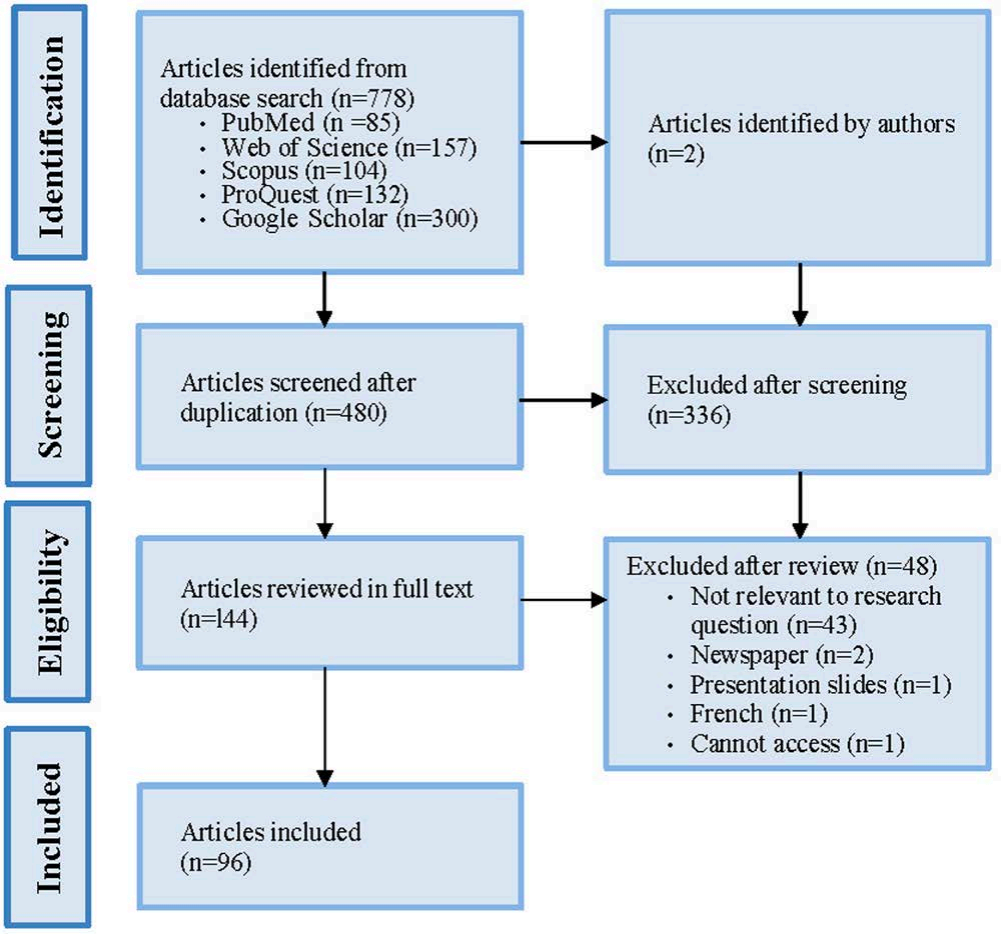
Flow chart of selected studies.

A total of 778 records were retrieved through database searches supplemented by two published articles known to the authors. Figure 1 shows the PRISMA flow diagram. After removing duplicates, 480 records underwent title and abstract screening, leading to the exclusion of 336 records. Full-text review was conducted on 144 records, resulting in the exclusion of 48 records. Finally, 96 studies were included (Supplementary Tables 1–3; http://links.lww.com/EE/A321).

Table 1 summarizes the results of the 96 included studies. Most of the studies (84.4%) were published in scientific journals, while others (15.6%) were gray literature, including government reports, conference abstracts, and student theses (Supplementary Table 1; http://links.lww.com/EE/A321). The published subset further breaks down as original research studies (64.2%), reviews (18.5%), and other publication types (17.3%), such as letters to the editor and technical reports. North America and the East Asia and the Pacific region were the main study areas where the studies were conducted (27.1% and 22.9%, respectively). Most studies examine the entire population (76%), while others either also examine or solely focus on specific subgroups (24%).

**Table 1.** Descriptive characteristics of selected studies (N = 96)

	No. studies (%)
Publication type	
Journal publication	81 (84.4)
Research	52 (64.2)
Review	15 (18.5)
Others	14 (17.3)
Gray literature	15 (15.6)
Study region	
East Asia and the Pacific	22 (22.9)
Latin America and the Caribbean	10 (10.4)
Middle East and North Africa	1 (1.0)
North America	26 (27.1)
South Asia	18 (18.8)
Sub-Saharan Africa	5 (5.2)
Not region-specific^a^	14 (14.6)
Study population	
All population	73 (76.0)
Children (≤5 years old)^b^	15 (15.6)
Elderly (>50 years old)^b^	4 (4.2)
Evacuee and relief worker	10 (10.4)
Others^c^	6 (6.3)

a “Not region-specific” refers to studies that reported results for more than one World Bank region or at a global scale.

b Age definitions for “Children” and “Elderly” differ across studies; however, all fall within the age ranges specified in this table.

c “Others” includes smallholder farmers, females, pregnant women, university students, and adults between 18 and 64 years old.

For further analysis, we identified 23 epidemiological studies that satisfy the criteria of having a clear study objective of quantifying the association between tropical cyclones and diarrheal diseases. Sixty percent of these epidemiological studies analyzed a single tropical cyclone event, while the rest examined the impact of multiple tropical cyclones (Table 2). In single tropical cyclone studies, three studies^32–34^ assessed the impact of Typhoon Haiyan on diarrheal diseases in the Philippines. Among the studies examining multiple tropical cyclones, 77.8% analyzed approximately 10 years of data, while 22.2% utilized more than 20 years of data (Supplementary Table 3; http://links.lww.com/EE/A321). Most of the epidemiological studies defined tropical cyclone exposure as an event with a fixed timeframe (69.6%). Twenty-six percent of studies used maximum wind speed, 13% used flooding, and 22% were based on rainfall. Event-based studies are often defined in combination with additional factors, such as flood,^35^ rain,^36^ or both wind and rain.^37^ Additionally, three studies considered multiple criteria to define tropical cyclone exposure.^15,38,39^

**Table 2.** Outcome variable in the epidemiological studies (n = 23)

	No. studies (%)
Tropical cyclone event	
Single	14 (60.9)
Multiple	9 (39.1)
Exposure definition	
Event based	16 (69.6)
Wind speed	6 (26.1)
Flooding	3 (13.0)
Rainfall	5 (21.7)
Health outcome	
Surveillance	10 (43.5)
Healthcare facility visits	7 (30.4)
Hospital admission	4 (17.4)
Self-report survey	3 (13.0)
Multiple outcomes	1 (4.3)
Pathogen	
All-cause (not pathogen-specific)	14 (60.9)
Pathogen-specific (including one or more pathogens)	9 (39.1)
Study design	
Pre-post comparison	14 (60.9)
Interrupted time series/difference-in-difference	5 (21.7)
Case-crossover	4 (17.4)
Direction of association	
Positive	15 (65.2)
Mixed^a^	3 (13.0)
Negative	1 (4.3)
Not significant	4 (17.4)

a “Mixed” direction of association indicates variability in results, which may differ based on pathogen-specific outcomes or regional variations within the studies (details provided in Supplementary Table 3; http://links.lww.com/EE/A321).

Almost half of the epidemiological studies (43.5%) analyzed cases of diarrheal diseases reported from existing surveillance systems, while the others analyzed cases from healthcare facility visits (30.4%) and self-report surveys (13.0%) (Table 2). Sixty-one percent of epidemiological studies analyzed only all-cause diarrheal diseases. For pathogen-specific studies, shigellosis stands out as the most studied diarrheal disease followed by cholera and typhoid (Supplementary Table 3; http://links.lww.com/EE/A321). Other studied diseases were paratyphoid fever, salmonellosis, *Escherichia coli* infection, giardiasis, and cryptosporidiosis. Three Chinese studies grouped viral diseases, such as rotavirus and norovirus infections, and protozoan diseases, like giardiasis and cryptosporidiosis, under the broader category of “other infectious diseases,” without differentiation (see note of Supplementary Table 3; http://links.lww.com/EE/A321).^15,38,40^

The most common study design was pre-post comparison without controlling for time-varying factors (60.9%). Other designs included interrupted time series and differences-in-difference (21.7%), which accounted for time-varying factors and control groups, and case-crossover design (17.4%). Over half of the studies (65.2%) found positive associations, showing an increased risk of diarrheal diseases linked to tropical cyclones. One study (4.3%) reported negative outcomes, indicating a decrease in diarrheal diseases, 13% presented mixed results varying by region or pathogen subgroups, and 17.4% found no significant associations.

### Review of transmission pathways

**Figure 2.**
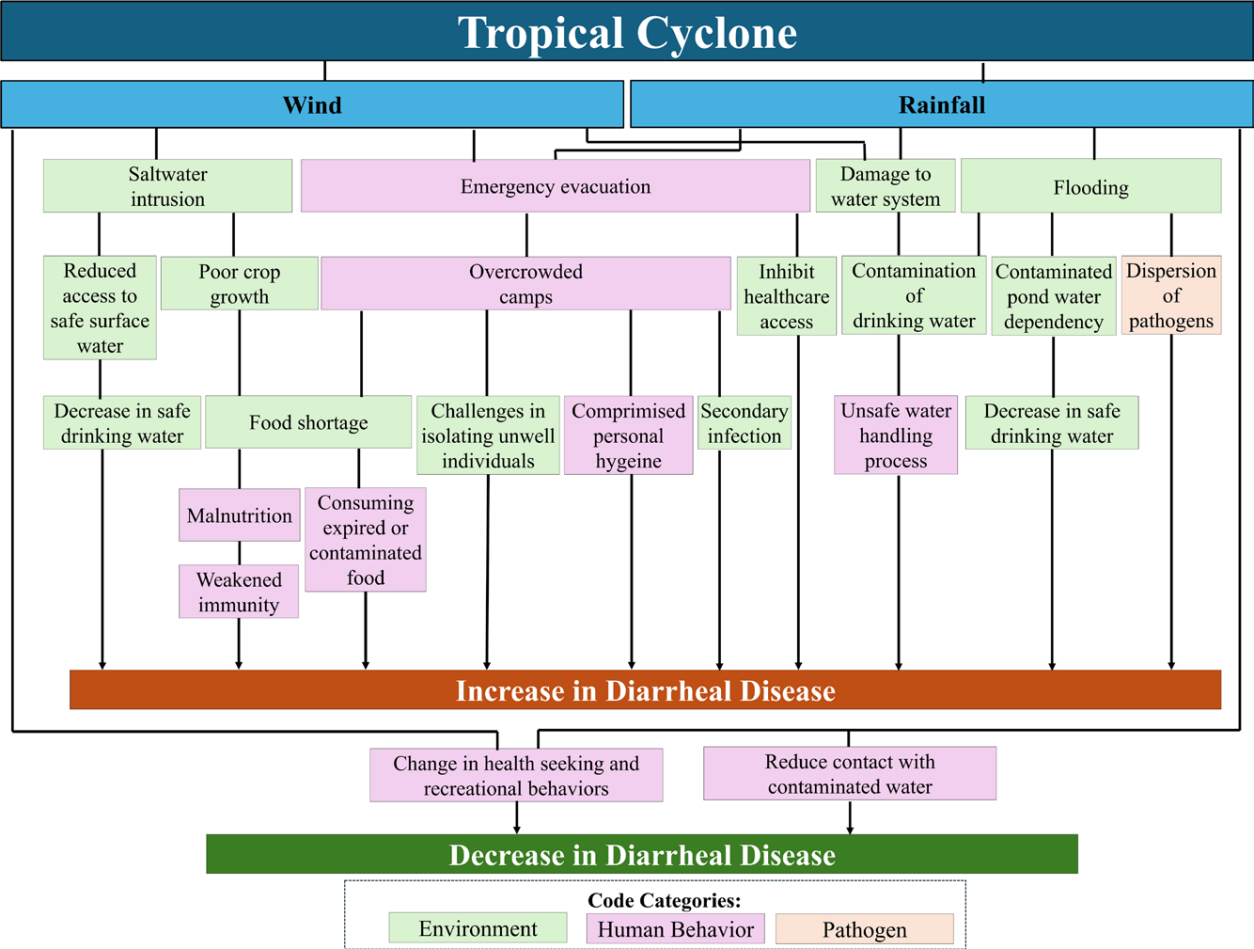
Transmission pathways for tropical cyclones to diarrheal diseases. Figure was created based on the thematic analysis of 30 studies that specifically examined transmission pathways linking tropical cyclones to diarrheal diseases.

Of the 96 articles included, 30 discussed the transmission pathways between tropical cyclones and diarrheal diseases; these discussions were used for thematic analysis, and the results are presented in Figure 2. Two main themes were identified: wind and rainfall, both directly caused by tropical cyclones. Subthemes illustrate the effects driven by these factors and are categorized into environment (green), human behavior (pink), and pathogen (orange).

#### Wind

Strong winds from tropical cyclones contribute to saltwater intrusion in coastal regions through storm surges, where saline water infiltrates freshwater reservoirs such as estuaries and aquifers. This intrusion elevates salinity levels, degrading groundwater quality and leading to reduced access to safe surface water and a decrease in safe drinking water. In coastal Bangladesh, salinity affects local sources such as rivers and ponds, forcing communities to travel long distances for alternatives that are often dirty, turbid, and odorous, harboring pathogens that heighten the risk of diarrhea risks. Furthermore, saltwater intrusion raises soil and water salinity, disrupting agriculture by negatively impacting soil quality and leading to poor crop growth, which causes food shortages and subsequently malnutrition. With weakened immunity due to food scarcity, affected populations become more vulnerable to diarrhea-causing pathogens.

#### Rainfall

Heavy rainfall leads to flooding that could cause contamination of drinking water and facilitate the dispersion of pathogens from excreta in soil to surface. Limited access to tube wells during floods increases contaminated pond water dependency and causes a decrease in safe drinking water, thereby facilitating the transmission of diarrheal diseases.

#### Wind and rainfall

Strong winds and heavy rainfall generated by tropical cyclones often lead to widespread damage to the water system, disrupting access to clean and safe drinking water and frequently necessitating emergency evacuation to protect affected populations from life-threatening hazards.

Damage to water system leads to the contamination of drinking water. Breaches in water pipelines allow contaminants to enter the water supply. Municipal water systems, compromised during tropical cyclone events, have been identified as likely sources of contaminated water. In addition to the contamination itself, unsafe water handling practices, such as failing to boil or chlorinate drinking water, further exacerbate the problem by increasing the likelihood of consuming unsafe water.

Emergency evacuation contributes significantly to the rise in diarrheal diseases following tropical cyclones by inhibiting healthcare access and creating overcrowded camps. These camps often lack sanitary facilities and clean water, leading to compromised personal hygiene. Overcrowding also poses challenges in isolating unwell individuals and increases the risk of secondary infections among relief workers and healthcare professionals. Additionally, food shortages force people to resort to consuming expired or contaminated food, further exacerbating the spread of diarrheal diseases.

#### Decrease in diarrheal disease

Tropical cyclones may reduce diarrheal disease cases as people stay indoors and avoid high-risk areas, thereby reducing contact with contaminated water. Tropical cyclone events also prompt a change in health-seeking behaviors, with strong winds often discouraging individuals from seeking medical treatment, potentially leading to underreporting of actual cases. Changes in recreational behaviors, such as avoiding public swimming pools and rivers during storm weeks, may further decrease the transmission of waterborne pathogens such as *Giardia*, which is commonly spread through public pools.

## Discussion

This scoping review examined the relationship between tropical cyclones and diarrheal diseases, with a specific emphasis on epidemiological studies and transmission pathways. We found valuable insights into several key aspects, including the region of the study, the definition of exposure and outcomes, and the study design employed to investigate the effect of tropical cyclones on diarrheal diseases.

### Study area and population

East Asia and the Pacific and North America are the most studied regions given that they receive the greatest number of tropical cyclones annually and have considerable populations affected. The United States of America, Bangladesh, and the Philippines are the most studied countries. Some low- and middle-income countries that have considerable diarrheal disease incidence^41^ and are affected by tropical cyclones without any study are Madagascar, Belize, Mexico, and Vietnam. Moreover, only a few studies have considered vulnerable subpopulations like children and displaced individuals/households. Future studies would benefit from expanding to other vulnerable locations and subpopulations for more targeted policies and disaster risk and management efforts.

### Outcome variables

All reviewed epidemiological studies focus on morbidity, with variability in defining diarrheal diseases. Some studies utilized ICD codes,^34,35,37^ others rely on self-reported symptoms,^42–44^ and Chinese studies^38,40^ adopt their national definitions. ICD codes offer standardized data comparison across hospitals, regions, and countries, facilitating cross-study analyses; however, some caution should be noted on ICD coding variability across countries.^45^ Self-reported surveys, though susceptible to recall bias or misinterpretation, can capture a broader spectrum of diarrheal cases, including mild or self-limiting episodes that do not require treatment at healthcare facilities. For instance, a US study found that only 20% of individuals with diarrheal illness seek medical attention.^46^ Consequently, healthcare facility data may underestimate the true community burden when used as a proxy for community-level incidence.^47^

Among disease-specific studies, the majority focus on bacterial diseases such as cholera, paratyphoid fever, nontyphoidal salmonellosis, shigellosis, and *E. coli* infections. Some studies have also investigated protozoan diseases such as giardiasis and cryptosporidiosis.^39,48^ Pathogen-specific analysis revealed mixed results. Kang et al^40^ reported no significant differences in weekly cases of dysentery, typhoid, and paratyphoid before and after tropical cyclones. However, weekly cases of “other infectious diarrhea” were significantly higher following most tropical cyclones. Lynch et al^39^ found that cryptosporidiosis cases increased during the storm week, with effects weakening in subsequent weeks. In contrast, *E. coli* infections and shigellosis both decreased during the storm week, while salmonellosis and giardiasis showed no significant association with storm exposure (details of pathogen-specific effect are provided in Supplementary Table 3; http://links.lww.com/EE/A321).

Mixed results from pathogen-specific analysis underscore the importance of dissecting differences in risk following a tropical cyclone to better assess and address public health impacts, but such studies face significant challenges. For example, hospital admissions or healthcare facility data often lack pathogen-specific information in discharge records, as many diagnoses are made without laboratory testing. Even when testing is conducted, pathogen details may be inaccurately recorded.^46^ Additionally, pathogen-specific studies are frequently constrained by smaller sample sizes, further limiting their scope and reliability.

Future research should explore mortality to gain critical insights into severe cases of diarrheal diseases. Conducting more pathogen-specific analyses is also valuable for identifying which pathogens proliferate after tropical cyclones. However, it is important to consider that this approach faces challenges related to underreported and misclassified pathogen data when interpreting the results.^49^ Additionally, if data are available, we recommend that future studies adopt ICD-10 codes A00–A09 to accurately represent diarrheal diseases caused by infectious enteric pathogens.^50^

### Exposure variables

Our review revealed substantial variability in exposure definitions across multiple cyclone studies, with definitions based solely on the time of the event, wind speed, rainfall, flooding, or a combination of these variables. Most studies relied on single-metric definitions, which, while straightforward, often fail to capture the diverse impacts of cyclone-related hazards such as wind and rain, potentially introducing biases.^51^

Wind-based exposure offers a straightforward definition, as the World Meteorological Organization has already established predefined tropical cyclone categories using maximum sustained winds.^52^ However, the accuracy of wind data sources remains a concern. Four studies that used wind speed as an exposure metric relied on weather station data from national meteorological agencies.^37,38,40,53^ Two studies mentioned limitations such as spatial granularity, with exposure data often based on the nearest available weather station rather than directly within the path of the tropical cyclone, leading to potential inaccuracies.^37,40^ One study highlighted interpolation challenges in regions with sparse monitoring stations, such as Eastern Taiwan, potentially compromising data reliability.^53^ An often-overlooked limitation of using weather station data is the risk of equipment damage during extreme events, potentially impacting data completeness and accuracy.^54^ Satellite data present a possible solution, offering broader spatial coverage and the ability to capture track and peak wind speed more accurately.^49^

Event-based approaches can effectively select strong tropical cyclones with descriptions of heavy rain, wind, and flooding. For example, using the Emergency Events Database (EM-DAT) allows the selection of storms with flooding. However, event-based methods may oversimplify cyclone impacts across diverse locations. To address this, studies can utilize tropical cyclone track data with wind field models to estimate local wind conditions and rainfall analysis within a certain radius of the cyclone’s eye to categorize regional rainfall, enabling analysis at subnational or smaller geographical scales.^55^

In addition, event-based methods often fail to capture the specific impacts of individual tropical cyclone hazards on diarrheal diseases. Analyzing hazards in distinct categories and their combinations can provide valuable insights. For example, one study categorized wind and rainfall exposure, showing that a rain-low/wind-low pairing during the storm week increased cryptosporidiosis cases more than a high-rain/high-wind pairing.^39^ This highlights how different tropical cyclone characteristics uniquely influence health risks. We recommend future research explore varying thresholds for categorizing cyclone-related hazards and examine the combined effects of multiple exposures.

### Study design

Most studies utilized a pre-post comparison, which is a rapid and convenient method to evaluate the effect of a major event on an outcome by comparing the pre-event period (control) with the postevent period. However, this approach typically lacks a concurrent control group, which can introduce limitations affecting internal validity.^56^ First is the arbitrary selection of pre and post timeframe. For example, three studies analyzed the impacts of typhoon Haiyan on diarrheal diseases in the same province, and selected timeframes lasting 1 week, 6 weeks, or 2 months before and after the date of typhoon landfall.^32–34^ Varying timeframes can yield different findings, as the transmission of diarrheal diseases takes time to establish and peak. It is crucial to consider the health system and socioeconomic conditions of the study location when selecting appropriate timeframes and lag structures. Second, is the lack of consideration for interannual trends, seasonality, and other confounders such as rainfall and flooding. These temporal and time-varying parameters can affect the results, especially in time series analysis and longer pre-post timeframes.

To address certain limitations, using advanced designs such as difference-in-difference^57^ or controlled interrupted time series^58^ can provide more robust measurements of the association between tropical cyclone events and diarrheal diseases. These designs offer improved control over confounding variables, such as temporal variations and population changes, as well as lag effects. Our scoping review showed that more recent studies have begun to adopt these designs.^35,59^ However, it is important to note that these designs are limited to analyzing the effects of individual tropical cyclone events.

In regions prone to frequent tropical cyclones, measuring the health impact of multiple tropical cyclone events would be important. This can be accomplished using the case-crossover design, which functions as a self-controlled study design where each case serves as its own control. Our review identified several studies that applied this approach, utilizing either unidirectional, bidirectional, or time-stratified designs (refer to Supplementary Table 3; http://links.lww.com/EE/A321). Studies employing the unidirectional design typically select the days before the case period as the control period, but the approach is subject to biases because of the nonindependent selection of controls, as well as biases associated with trends and seasonal patterns in the exposure.^60^ In contrast, the time-stratified design, applied in one^39^ of the four case-crossover studies,^38–40,53^ effectively addresses these issues. By selecting control periods from both directions around the event and restricting them to the same day of the week, month, and year, the time-stratified approach minimizes biases related to trends, seasonality, and other time-varying confounders, providing a more reliable assessment of the health impact associated with tropical cyclones.^61^

Future studies examining the relationship between tropical cyclones and diarrheal diseases should thoughtfully select study designs, particularly when using time series data from surveillance systems or electronic health records. A thorough review of tropical cyclone exposure characteristics, the specific nature of diarrheal diseases, and spatiotemporal dimensions is crucial to inform the most suitable study design and analytical approach.

### Transmission pathways

In the thematic analysis, we identified key transmission pathways linking tropical cyclones and diarrheal diseases; however, further research is needed to strengthen these findings. Effect modifiers such as income and geographic setting could provide valuable insights into these pathways. For example, the study by Huang et al^8^ found higher hospitalization risks in areas with greater relative deprivation index levels – an index measuring global deprivation and poverty through sociodemographic and satellite data – highlighting the role of socioeconomic disparities in shaping vulnerability. These disparities can influence transmission pathways by affecting access to clean water, sanitation, and healthcare resources, as well as the capacity to respond to and recover from cyclone-induced hazards. Geographic differences between urban and rural areas also play a role. While two US studies in our review found no significant risk differences, greater disparities in other countries may produce contrasting results.^35,39^ Investigating these modifiers can help clarify how tropical cyclones influence disease transmission across diverse contexts.

A limitation of our study is the focus on human health research, which may overlook valuable insights from environmental studies on water and soil contamination post cyclone. This focus contributes to the lack of specific explanations in the suggested transmission pathways, leaving critical gaps in understanding how tropical cyclones impact diarrheal diseases. We propose cooperation between environmental sampling studies, such as post cyclone water quality assessments, and health outcome studies to pinpoint direct transmission pathways and enable more effective policymaking.^62^

## Conclusions

Tropical cyclones are significant natural disasters that impact human health by intensifying climate-sensitive diarrheal diseases. In existing literature, the lack of standardized definitions for outcomes, exposures, and methodologies hinders the comparability of results. To enhance a comprehensive understanding of the health impacts of tropical cyclones, we propose several key recommendations for future research. First, study designs should incorporate advanced analytical approaches, such as difference-in-difference or controlled interrupted time series for individual tropical cyclone events, and case-crossover designs for studies involving multiple events. Second, future studies should examine how varying tropical cyclone exposure definitions influence results to determine the most appropriate metrics and explore the combined effects of multiple exposures. Finally, we encourage conducting studies integrating health data with environmental sampling to better understand the transmission pathways between tropical cyclones and diarrheal diseases.

## Conflicts of interest statement

The authors declare that they have no conflicts of interest with regard to the content of this report.

## Supplementary Material


