# Supplementary material for: A scoping review and thematic analysis of the effects of tropical cyclones on diarrheal diseases
Source: Environ Epidemiol. 2025 Jan 9;9(1):e366. doi: 10.1097/EE9.0000000000000366 (PMC11723668; doi:10.1097/EE9.0000000000000366)
Supplement: Supplementary file 1 [file ee9-9-e366-s001.pdf]

[Environmental Epidemiology]

Supplementary Material for

[A Scoping Review and Thematic Analysis of the Effects of Tropical Cyclones on Diarrheal Diseases]

[Szu Yu Lin[1], Paul LC Chua[1], Lei Yuan[1], Nasif Hossain[1], Jinyu He[3], Lisa Yamasaki[1,4], Lina Madaniyazi[2], Chris Fook Sheng Ng[1,2], Aurelio Tobias[2,5], Masahiro Hashizume[1,2]]

[1 Department of Global Health Policy, Graduate School of Medicine, The University of Tokyo, Tokyo, Japan.
2 School of Tropical Medicine and Global Health, Nagasaki University, Nagasaki, Japan.
3 Center for Climate Change Adaptation, National Institute for Environmental Studies, Tsukuba, Japan.
4 Center Hospital of the National Center for Global Health and Medicine, Tokyo, Japan.
5 Institute of Environmental Assessment and Water Research (IDAEA), Spanish Council for Scientific Research (CSIS), Barcelona, Spain.]

Supplementary Text 1. Literature search strategy

- PubMed search strategy:

("tropical cyclone"[All Fields] OR ("cyclonic storms"[MeSH Terms] OR ("cyclonic"[All Fields] AND "storms"[All Fields]) OR "cyclonic storms"[All Fields] OR "hurricane"[All Fields] OR "hurricanes"[All Fields] OR "hurricane s"[All Fields]) OR ("cyclonic storms"[MeSH Terms] OR ("cyclonic"[All Fields] AND "storms"[All Fields]) OR "cyclonic storms"[All Fields] OR "typhoon"[All Fields] OR "typhoons"[All Fields]) OR "tropical storm"[All Fields] OR "tropical depression"[All Fields] OR "cyclon*"[All Fields] OR "cyclonic storm"[All Fields]) AND ("diarrh*"[All Fields] OR "enteric infection"[All Fields] OR "gastroenteri*"[All Fields] OR "waterborne disease*"[All Fields])

- Web of Science search strategy:

(ALL=(tropical cyclone) OR ALL=(hurricane) OR ALL=(typhoon) OR ALL=(tropical storm) OR ALL=(tropical depression) OR ALL=(cyclon*) OR ALL=(cyclonic storm)) AND (ALL=(diarrh*) OR ALL=(enteric infection) OR ALL=(gastroenteri*) OR ALL=(waterborne disease*))

- Scopus search strategy:

TITLE-ABS ("tropical cyclone" OR hurricane OR typhoon OR "tropical storm" OR "tropical depression" OR cyclon*) AND TITLE-ABS (diarrh* OR "enteric infection" OR gastroenter* OR "waterborne disease") AND (LIMIT-TO (LANGUAGE , "English"))

- Google Scholar (first 300 items) search strategy:

"tropical cyclone"|hurricane|typhoon|"tropical storm"|"tropical depression"|cyclone* diarrhea*|"enteric infection"|gastroenter*|"waterborne disease"

- Proquest search strategy:

((summary(tropical cyclone) OR summary(hurricane) OR summary(typhoon) OR summary(tropical storm) OR summary(tropical depression) OR summary(cyclone)) AND la.exact("ENG")) AND ((summary(diarrhea) OR summary(enteric infection) OR summary(gastroenteritis) OR summary(waterborne disease)) AND la.exact("ENG"))

Supplementary Table 1. Characteristics of all studies included in the scoping review

First Author	Year	Publication Type	Study Region	Study Population
Vèlimirovic & Subramanian	1972	original research paper	The Phillippines	all
Bissell	1983	review	Dominican Republic	all
Siddique et al.	1989	original research paper	Bangladesh	all
Bennish & Ronsmans	1992	special article	Bangladesh	all
Lee et al.	1993	original research paper	USA	all
Sheppa et al.	1993	original research paper	USA	all
Alam et al.	1993	report	Bangladesh	all
Matsuda	1993	original research paper	Bangladesh	all
Simeon et al.	1993	original research paper	Jamaica	children
Quinn et al.	1994	report	USA	children
Campanella	1999	original research paper	Nicaragua	all
Moll et al.	2001	original research paper	USA	children
Guill & Shandera	2001	original research paper	Honduras	all
Setzer & Domino	2004	original research paper	USA	all
Kleinau et al.	2004	strategic report	Global	all
CDC	2005	report	USA	all
CDC	2005	report	USA	evacuee
Morantz	2005	practice guideline brief	USA	evacuee, relief worker
CDC	2005	report	USA	evacuee
CDC	2005	report	USA	all
Conly & Johnston	2005	notes	Global	all
Arnold	2006	Editor's corner, other	USA	all
Chui et al.	2006	ISEE conference abstract	USA	elderly
Partridge et al.	2006	original research paper	Federated States of Micronesia	relief worker
Christenson & Bernard	2006	perspective/opinion	Puerto Rico	all

Moll et al.	2007	original research paper	USA	children
Rath et al.	2007	original research paper	USA	children
Yee et al.	2007	original research paper	USA	evacuee
Khuntia et al.	2008	original research paper	India	all
Cavey et al.	2009	special report	USA	evacuee
Tyebally	2009	abstract	Myanmar	all
Murthy & Christian	2010	review	Global	all
Bhunia & Ghosh	2011	original research paper	India	all
Panda et al.	2011	original research paper	India	all
Paul et al.	2011	original research paper	Bangladesh	all
Lemonick	2011	review	Global	all
Lin et al.	2011	original research paper	Guatemala	all
Myint et al.	2011	original research paper	Myanmar	all
Alhinai	2011	original research paper	Oman	all
Rabbani et al.	2012	original research paper	Bangladesh	all
Kouadio et al.	2012	review	Global	all
Ha et al.	2012	ISEE conference abstract	South Korea	all, elderly
Dolhun	2013	Letter to the editor, other	The Phillippines	all
Kim et al.	2013	original research paper	South Korea	all, elderly
Adhisivam et al.	2014	scientific letter	India	all, children, parent
Kim et al.	2014	review	Global	all
Ahdoot et al.	2015	technical report	USA	children
Gaither et al.	2015	original research paper	USA	evacuee
Ridpath et al.	2015	original research paper	USA	evacuee
Fredrick et al.	2015	original research paper	India	all, elderly, female
Savage et al.	2015	original research paper	The Phillippines	all
Ventura et al.	2015	original research paper	The Phillippines	all
Deng et al.	2015	original research paper	China	all
Kang et al.	2015	original research paper	China	all, children

Wang et al.	2015	Letter to the editor, other	China	all, children
Davies et al.	2015	original research paper	Cambodia	all
Bloom et al.	2016	original research paper	USA	all, children
Munnaf	2016	thesis	Bangladesh	all
Chang et al.	2016	original research paper	The Phillippines	all, children
Sato et al.	2016	original research paper	The Phillippines	female, pregnant women
Kousky	2016	review	Global	children
Levy et al.	2016	review	Global	all
Na et al.	2016	original research paper	South Korea	all
Salazar et al.	2017	original research paper	The Phillippines	all, children
Saulnier & Ribacke	2017	review	Global	all
Khan et al.	2017	original research paper	Haiti	all
Zheng et al.	2017	original research paper	China	all
Adedokun	2018	thesis	USA	university student
Van Loenhout et al.	2018	original research paper	The Phillippines	all
Etheart et al.	2018	abstract	Haiti	all
Haque et al.	2019	original research paper	Bangladesh	all
Varo & López	2019	original research paper	Global	all
Hulland et al.	2019	original research paper	Haiti	children
Ma & Jiang B.	2019	review	China	all
Cambaza et al.	2019	short communication	Mozambique	all
Chen & Azman A. S.	2019	perspective piece	Mozambique	all
Loebach & Korinek	2019	original research paper	Nicaragua	evacuee
Jacot Des Combes	2019	original research paper	Fiji	all
Aka	2020	thesis	Bangladesh	evacuee
Lequechane & Mahumane	2020	Letter to the editor	Mozambique	all
Kraay et al.	2020	review	Global	all
Ramesh et al.	2021	abstract	USA	all
Ramesh et al.	2021	original research paper	USA	all

Giribabu et al.	2021	original research paper	India	all
Rafa et al.	2021	original research paper	Bangladesh	all
Talukder et al.	2021	review	Bangladesh, India, Malawi	smallholder farmer
Arpin et al.	2021	review	Global	children
Quist et al.	2022	original research paper	USA	all
Ramesh et al.	2022	original research paper	USA	all, adults (18-64), female
Kibria et al.	2022	review	Bangladesh	all
Chen et al.	2022	original research paper	Taiwan	all
Alcayna et al.	2022	review	Global	all
Kubo et al.	2022	original research paper	Mozambique	all
Lynch & Shaman	2023	original research paper	USA	all
Adriano et al.	2023	short communication	Malawi	all
Huang et al.	2023	review	Global	all

Supplementary Table 2. Characteristics, methods, and health outcomes of single TC studies included in the scoping review

First Author	Year	Name of TC	Exposure	Health Outcome	Pathogens	Study Design	Direction of Association[a]	Delayed Effect
Sheppa et al.	1993	Hurricane Hugo	event	healthcare facility visits	not pathogen-specific	pre-post comparison	not significant	NA
Simeon et al.	1993	Hurricane Gilbert	event	self reported survey	not pathogen-specific	pre-post comparison	positive	NA
Setzer & Domino	2004	Hurricane Floyd	event	healthcare facility visits	Cryptosporidiosis, Giardia lamblia	difference in difference	not significant	NA
Rath et al.	2007	Hurricane Katrina	event	self reported survey	not pathogen-specific	pre-post comparison	positive	NA
Myint et al.	2011	Cyclone Nargis	event	surveillance	not pathogen-specific	pre-post comparison	positive	NA
Alhinai	2011	Cyclone Gonu	event	healthcare facility visits	not pathogen-specific	pre-post comparison	positive	NA
Bloom et al.	2016	Hurricane Sandy	event	healthcare facility visits, hospital admission	not pathogen-specific	pre-post comparison	negative	13 days after landfall
Chang et al.	2016	Typhoon Haiyan	event	hospital admission	not pathogen-specific	pre-post comparison	positive	Incidence peaked during impact phrases then decreased in post-impact phase
Salazar et al.	2017	Typhoon Haiyan	event	surveillance	not pathogen-specific	pre-post comparison	positive	Risk within first 2 months higher than risk more than 2 months
Van Loenhout et al.	2018	Typhoon Haiyan	event	hospital admission	not pathogen-specific	pre-post comparison	positive	Drop in hospital admission on the day of tropical cyclon;<br><br>In one hospital, admission surpassed

								baseline after 2 weeks.
Hulland et al.	2019	Hurricane Matthew	event	surveillance	Cholera	interrupted time series	Mixed (vary by sub-national level)	Immediate increase followed by significant decline
Loebach & Korinek	2019	Hurricane Mitch	event	self reported survey	not pathogen-specific	pre-post comparison	positive	Post tropical cyclone
Ramesh et al.	2021	Hurricane Harvey	flooding	healthcare facility visits	not pathogen-specific	interrupted time series	positive	Increased risk during flood period and decreases
Ramesh et al.	2022	Tropical Storm Imelda	flooding	healthcare facility visits	not pathogen-specific	interrupted time series	positive	2 months after landfall

Note: [a]We defined the direction of association as positive if a significantly positive result was observed at any point during the study period.

Supplementary Table 3. Characteristics, methods, and health outcomes of multiple TC studies included in the scoping review

First Author	Year	Study Length	Exposure Definition	Health Outcome	Pathogens	Study Design	Direction of Association[a]	Delayed Effect
Vèlimirovic & Subramanian	1972	2 years	event	surveillance	Cholera, Typhoid fever	pre-post comparison	not significant	NA
Kim et al.	2013	7 years	event (wind + rainfall)	hospital admission	not pathogen-specific	pre-post comparison	positive	Highest RR at lag 0
Deng et al.	2015	7 years	multiple criteria (wind + rainfall)	surveillance	Bacillary dysentery, other infectious diarrhea[b]	case-crossover	positive	For Bacillary dysentery highest impact at lag 6;<br><br>For other infectious diarrhea highest impact at lag 5
Kang et al.	2015	7 years	wind	surveillance	Bacillary dysentery, Paratyphoid fever, Typhoid fever, other infectious diarrhea[b]	case-crossover	mixed<br><br>(No significant difference in dysentery, typhoid, and paratyphoid before and after tropical cyclones;<br><br>Significant differences in other infectious diarrhea after tropical cyclones)	For infectious diarrhea other infectious diarrheal diseases:<br><br>Tropical depression impact largest effect at lag 1 for total population<br><br>Tropical storm, severe tropical storm and typhoon largest effect at lag 0 for total population
Na et al.	2016	9 years	event (rainfall)	surveillance	Shigellosis, Typhoid, Paratyphoid fever,	pre-post comparison	not significant	NA
Zheng et al.	2017	7 years	multiple criteria (wind + rainfall)	surveillance	Bacillary dysentery, Paratyphoid fever, Typhoid fever,	pre-post comparison	positive	NA

					other infectious diarrhea[b]			
Quist et al.	2022	4 years	event (flood)	healthcare facility visits	not pathogen-specific	interrupted time series	positive	Increased pooled rate ratio for Hurricane Matthew and Florence three weeks after the event
Chen et al.	2022	22 years	wind	surveillance	Shigellosis	case-crossover	positive	Significant increase at lag 11
Lynch & Shaman	2023	23 years	multiple criteria (wind + rainfall)	surveillance	Cryptosporuduisis, Giardiasis, E.coli, Salmonella, Shigellosis	case-crossover	Mixed<br><br>(Cryptosporuduisis positively associated with storm exposure;<br><br>Salmonella and Giardiasis not significantly associated with storm exposure)	Cryptosporuduisis increase in the week of storm, effects becomes weaker as lag weeks increase<br><br>E.coli decrease on the week of storm<br><br>Shigellosis decrease in the week of storm

Note: [a]We defined the direction of association as positive if a significantly positive result was observed at any point during the study period.

[b]Other infectious diarrhea, as defined by the Chinese National Notifiable Disease Surveillance System (NDSS), includes a variety of common infections. These encompass salmonella enteritis, *enteropathogenic Escherichia coli (E. coli) enteritis*, *enteropathogenic vibrio enteritis*, *Yersinia enterocolitica enteritis*, rotavirus enteritis, Norovirus gastroenteritis, enteric adenovirus enteritis, cryptosporidiosis, and giardiasis.

Supplementary Table 4. Thematic analysis

First Author	Year	Main Theme(s)	Sub Theme(s)
Bissell	1983	Wind; Rainfall	Emergency evacuation
Siddique et al.	1989	Rainfall	Contaminated pond water
Matsuda et al.	1993	Wind; Rainfall	Change in health-seeking and recreational behaviors
Morantz et al.	2005	Wind; Rainfall	Emergency evacuation;<br>Secondary infection
CDC	2005	Wind; Rainfall	Emergency evacuation;<br>Challenges in isolating unwell individuals;<br>Compromised personal hygiene
Christenson et al.	2006	Wind; Rainfall	Emergency evacuation
Yee et al.	2007	Wind; Rainfall	Emergency evacuation;<br>Overcrowded camps;<br>Compromised personal hygiene
Murthy et al.	2010	Wind	Saltwater intrusion;<br>Weakened immunity
Bhunia et al.	2011	Wind	Damage to water system
Paul et al.	2011	Wind	Saltwater intrusion;<br>Reduced access to safe surface water
Kim et al.	2014	Wind	Damage in water system
Deng et al.	2015	Wind	Contamination of drinking water
Ventura et al.	2015	Wind	Unsafe water handling practices
Deng et al.	2015	Rainfall	Dispersion of pathogens;<br>Damage in water system
Ridpath et al.	2015	Wind; Rainfall	Emergency evacuation;<br>Challenges in isolating unwell individuals
Change et al.	2016	Rainfall	Contamination of drinking water
Munnaf et al.	2016	Wind; Rainfall	Saltwater intrusion;<br>Reduced access to safe surface water
Zheng et al.	2017	Rainfall	Flooding
Salazar et al.	2017	Wind; Rainfall	Emergency evacuation
Saulnier et al.	2017	Wind; Rainfall	Emergency evacuation
Adedokun et al.	2018	Wind; Rainfall	Emergency evacuation;<br>Food shortages;

			Consuming expired or contaminated food
Varo et al.	2019	Wind	Damage to water system
Loebach et al.	2019	Wind; Rainfall	Saltwater intrusion;<br>Emergency evacuation;<br>Overcrowded camps;<br>Malnutrition
Varo et al.	2019	Wind; Rainfall	Emergency evacuation
Ramesh et al.	2021	Rainfall	Dispersion of pathogens
Talukder et al.	2021	Wind; Rainfall	Emergency evacuation;<br>Inhibite healthcare access
Alcayna et al.	2022	Wind; Rainfall	Emergency evacuation;<br>Flooding;<br>Malnutrition;<br>Compromised personal hygiene
Ramesh et al.	2022	Wind; Rainfall	Emergency evacuation;<br>Dispersion of pathogens;<br>Food shortages;<br>Consuming expired or contaminated food;<br>Compromised personal hygiene
Adriano et al.	2023	Wind	Saltwater intrusion;<br>Reduced access to safe surface water;<br>Poor crop growth;<br>Food shortages;<br>Compromised personal hygiene
Lynch et al.	2023	Wind; Rainfall	Emergency evacuation;<br>Change in health-seeking and recreational behaviors;<br>Reduce contact with contaminated water

List of studies included in the scoping review

Adedokun, A. O. (2018). Food safety of college students during and in the aftermath of Hurricane Harvey [MA thesis, Lamar University]. https://www.proquest.com/dissertations-theses/food-safety-college-students-during-aftermath/docview/2150714399/se-2

Adhisivam, B., Lukose, F., Subitha, L., & Bhat, B. V. (2014). Assessment of early impact of Cyclone Thane on physical and psychosocial environment. *Indian Journal of Pediatrics*, *81*(11), 1248. https://doi.org/10.1007/s12098-014-1372-0

Adriano, L. F., Nazir, A., & Uwishema, O. (2023). The devastating effect of cyclone Freddy amidst the deadliest cholera outbreak in Malawi: a double burden for an already weak healthcare system—short communication. *Annals of Medicine and Surgery*, *85*(7), 3761–3763. https://doi.org/10.1097/ms9.0000000000000961

Ahdoot, S., & Pacheco, S. E. (2015). Global climate change and children's health. *Pediatrics*, *136*(5), e1468–e1484. https://doi.org/10.1542/peds.2015-3233

Aka, S. H. (2020). A survey assessments of health and hygiene sustainability in the cyclone shelters situated in coastal zone of Bangladesh (Cox's Bazar sadar and Maheshkhali) [Thesis]. Brac University.

Alcayna, T., Fletcher, I., Gibb, R., Tremblay, L., Funk, S., Rao, B., & Lowe, R. (2022). Climate-sensitive disease outbreaks in the aftermath of extreme climatic events: A scoping review. *One Earth*, *5*(4), 336–350. https://doi.org/10.1016/j.oneear.2022.03.011

Alhinai, M. Y. (2011). Tropical Cyclone Gonu: Number of patients and pattern of illnesses in the primary health centers in A'Seeb area, Muscat, Sultanate of Oman. *Oman Medical Journal*, 223–228. https://doi.org/10.5001/omj.2011.56

Arnold, J. L. (2006). Disaster Myths and Hurricane Katrina 2005: Can Public Officals and the Media Learn to Provide Reponsible Crisis Communication during Disasters? *Prehospital and Disaster Medicine*, *21*(01), 1–3. https://doi.org/10.1017/s1049023x00003241

Arpin, E., Gauffin, K., Kerr, M. V., Hjern, A., Mashford‐ Pringle, A., Barros, A. J. D., Rajmil, L., Choonara, I., & Spencer, N. (2021). Climate Change and Child Health Inequality: A review of reviews. *International Journal of Environmental Research and Public Health*, *18*(20), 10896. https://doi.org/10.3390/ijerph182010896

Bennish, M. L., & Ronsmans, C. (1992). Health and nutritional consequences of the 1991 Bangladesh cyclone. *Nutrition Reviews*, *50*(4), 102–105. https://doi.org/10.1111/j.1753-4887.1992.tb01296.x

Bhunia, R., & Ghosh, S. (2011). Waterborne cholera outbreak following Cyclone Aila in Sundarban area of West Bengal, India, 2009. *Transactions of the Royal Society of Tropical Medicine and Hygiene*, *105*(4), 214–219. https://doi.org/10.1016/j.trstmh.2010.12.008

Bissell, R. A. (1983). Delayed-impact infectious disease after a natural disaster. *The Journal of Emergency Medicine*, *1*(1), 59–66. https://doi.org/10.1016/0736-4679(83)90010-0

Bloom, M. S., Palumbo, J., Saiyed, N. S., Lauper, U., & Lin, S. (2016). Food and waterborne disease in the greater New York City area following Hurricane Sandy in 2012. *Disaster Medicine and Public Health Preparedness*, *10*(3), 503–511. https://doi.org/10.1017/dmp.2016.85

Cambaza, E., Mongo, E., Anapakala, E., Nhambire, R., Singo, J., & Machava, E. (2019). Outbreak of cholera due to Cyclone Kenneth in northern Mozambique, 2019. *International Journal of Environmental Research and Public Health*, *16*(16), 2925. https://doi.org/10.3390/ijerph16162925

Campanella, N. (1999). Infectious diseases and natural disasters: the effects of Hurricane Mitch over Villanueva municipal area, Nicaragua. *Pubic Health Reviews*, *27*(4), 311–319.

Cavey, A. M. J., Spector, J. M., Ehrhardt, D., Kittle, T., McNeill, M., Greenough, P. G., & Kirsch, T. D. (2009). Mississippi's infectious Disease Hotline: A surveillance and education model for future disasters. *Prehospital and Disaster Medicine*, *24*(1), 11–17. https://doi.org/10.1017/s1049023x00006488

Chang, M. P., Simkin, D. J., De Lara, M. L., & Kirsch, T. D. (2016). Characterizing hospital admissions to a tertiary care hospital after Typhoon Haiyan. *Disaster Medicine and Public Health Preparedness*, *10*(2), 240–247. https://doi.org/10.1017/dmp.2015.165

Chen, N., Chen, Y., Wu, C., Chen, M., & Guo, Y. (2022). The impact of heavy precipitation and its impact modifiers on shigellosis occurrence during typhoon season in Taiwan: A case-crossover design. *Science of the Total Environment*, *848*, 157520. https://doi.org/10.1016/j.scitotenv.2022.157520

Chen, W. H., & Azman, A. S. (2019). Mitigating cholera in the aftermath of Cyclone Idai. *American Journal of Tropical Medicine and Hygiene*, *101*(5), 960–962. https://doi.org/10.4269/ajtmh.19-0285

Christenson, B. (2006). Oscar Costa-Mandry and posthurricane bacillary dysentery. *Clinical Infectious Diseases*, *42*(11), 1650–1651. https://doi.org/10.1086/503912

Chui, K. H., Castronovo, D. A., Jagai, J. S., Kosheleva, A. A., & Naumova, E. N. (2006). Gastroenteritis infections in the U.S. elderly and extreme weather events: exposures to Atlantic tropical storms of 1998–2002. *Epidemiology*. https://journals.lww.com/epidem/fulltext/2006/11001/gastroenteritis_infections_in_the_u_s__elderly_and.1283.aspx

Combes, H. (2019). Storm surges, heavy rain and strong wind: Impacts of Tropical Cyclone Winston in Fiji—Focus on Health. In *Springer eBooks* (pp. 185–196). https://doi.org/10.1007/978-3-030-23773-8_13

Conly, J., & Johnston, B. (2005). Natural disasters, corpses and the risk of infectious diseases. *Canadian Journal of Infectious Diseases & Medical Microbiology*, *16*(5), 269–270. https://doi.org/10.1155/2005/684640

Davies, G. I., McIver, L., Kim, Y., Hashizume, M., Iddings, S., & Chan, V. S. (2014). Water-Borne Diseases and Extreme Weather Events in Cambodia: Review of impacts and implications of climate change. *International Journal of Environmental Research and Public Health*, *12*(1), 191–213. https://doi.org/10.3390/ijerph120100191

Deng, Z., Xun, H., Zhou, M., Jiang, B., Wang, S., Guo, Q., Wang, W., Kang, R., Wang, X., Marley, G., & Ma, W. (2015). Impacts of tropical cyclones and accompanying precipitation on infectious diarrhea in cyclone landing areas of Zhejiang province, China. *International Journal of Environmental Research and Public Health*, *12*(2), 1054–1068. https://doi.org/10.3390/ijerph120201054

Dolhun, E. (2013). Aftermath of Typhoon Haiyan: The imminent epidemic of waterborne illnesses in Leyte, Philippines. *Disaster Medicine and Public Health Preparedness*, *7*(6), 547–548. https://doi.org/10.1017/dmp.2013.114

Etheart, M. D., Pierre, K., Jean-Charles, N., Destine, A., Andrécy, L. L., Barthelemy, N., Greiner, A., Giese, C., Juin, S., Hulland, E., Knipes, A., Adrien, P., Fitter, D. L., & Lafontant, D. (2018). A multidisciplinary joint-team efforts deployed for a cholera outbreak response post-hurricane Matthew in southern Haiti, October 2016. *International Journal of Infectious Diseases*, *73*, 273. https://doi.org/10.1016/j.ijid.2018.04.4037

Fredrick, T., Ponnaiah, M., Murhekar, M., Jayaraman, Y., David, J. K., Selvaraj, V., & Joshua, V. (2015). Cholera outbreak linked with lack of safe water supply following a tropical cyclone in Pondicherry, India, 2012. *Journal of Health, Population and Nutrition*, *33*(1), 31–38.

Gaither, J. B., Page, R., Prather, C., Paavola, F. G., & Garrett, A. L. (2015). Impact of a hurricane shelter viral gastroenteritis outbreak on a responding medical team. *Prehospital and Disaster Medicine*, *30*(4), 355–358. https://doi.org/10.1017/s1049023x15004872

Giribabu, D., Muvva, V. R., Joshi, N. K., & Rao, S. S. (2021). Assessment of epidemiological implications due to serial tropical cyclones in India: Introspecting the recent sanitation interventions. *Disaster Medicine and Public Health Preparedness*, *15*(2), 181–190. https://doi.org/10.1017/dmp.2019.129

Guill, C. K., & Shandera, W. X. (2001). The effects of Hurricane Mitch on a community in northern Honduras. *Prehospital and Disaster Medicine*, *16*(3), 166–171. https://doi.org/10.1017/s1049023x00025929

Ha, J., Kim, S., & Shin, Y. (Eds.). (2012). *Health impacts of typhoon and heavy rain disasters in South Korea* (23rd ed., Vol. 5S). *Epidemiology*. https://doi.org/10.1097/01.ede.0000417093.76239.c8

Haque, M. A., Alam, M. A., Moniruzzaman, S. M., & Hoque, M. M. (2019). The impact of climate change in the coastal areas of Bangladesh affected by Cyclone Bulbul. *Bangladesh Journal of Extension Education*, *31*, 13–27. http://bjee.com.bd/public/articles/Impact-of-Climate-Change-in-Coastal-Bangladesh-Affected-by-Cyclone-Bulbul.pdf

UNICEF (1993). Health effects of the 1991 Bangladesh Cyclone: Report of a UNICEF evaluation team. (1993). *Disasters*, *17*(2), 153–165. https://doi.org/10.1111/j.1467-7717.1993.tb01142.x

Huang, W., Gao, Y., Xu, R., Yang, Z., Yu, P., Ye, T., Ritchie, E. A., Li, S., & Lee, H. (2023). Health Effects of Cyclones: A Systematic Review and Meta-Analysis of Epidemiological Studies. *Environmental Health Perspectives*, *131*(8). https://doi.org/10.1289/ehp12158

Hulland, E., Subaiya, S., Pierre, K., Barthelemy, N., Pierre, J. S., Dismer, A., Juin, S., Fitter, D. L., & Brunkard, J. (2019). Increase in reported cholera cases in Haiti following Hurricane Matthew: An Interrupted Time series model. *American Journal of Tropical Medicine and Hygiene*, *100*(2), 368–373. https://doi.org/10.4269/ajtmh.17-0964

Illness surveillance and rapid needs assessment among Hurricane Katrina Evacuees - Colorado, September 1-23, 2005. (2006). In *Morbidity and Mortality Weekly Report* (55(09);244-247). https://www.cdc.gov/mmwr/preview/mmwrhtml/mm5509a7.htm

Infectious disease and dermatologic conditions in evacuees and rescue workers after Hurricane Katrina—Multiple States, August-September, 2005. (2005a). *JAMA*, *294*(17), 2158. https://doi.org/10.1001/jama.294.17.2158

Kang, R., Xun, H., Zhang, Y., Wang, W., Wang, X., Jiang, B., & Ma, W. (2015). Impacts of different grades of tropical cyclones on infectious diarrhea in Guangdong, 2005-2011. *PLOS ONE*, *10*(6), e0131423. https://doi.org/10.1371/journal.pone.0131423

Khan, R., Anwar, R., Akanda, S., McDonald, M. D., Huq, A., Jutla, A., & Colwell, R. R. (2017). Assessment of Risk of Cholera in Haiti following Hurricane Matthew. *American Journal of Tropical Medicine and Hygiene*, *97*(3), 896–903. https://doi.org/10.4269/ajtmh.17-0048

Khuntia, H. K., Samuel, A. K., Nayak, S. R., Sarangl, A. K., Mohanty, P., Kar, S., & Pal, B. (2008). Incidence, serotype, antibiogram and toxigenicity of vibrio cholerae during 2000, six month after the Super Cyclone, 1999 in Orissa, India. *Journal of Pure and Applied Microbiology*, *2*.

Kibria, G., Pavel, H. R., Miah, M. R., & Islam. (2022). Impacts of Climate Change in Bangladesh and its Consequences on Public Health. *Journal of Sustainability and Environmental Management*, *1*(3), 359–370. https://doi.org/10.3126/josem.v1i3.48002

Kim, K., Kabir, E., & Jahan, S. A. (2014). A review of the consequences of global climate change on human health. *Journal of Environmental Science and Health, Part A*, *32*(3), 299–318. https://doi.org/10.1080/10590501.2014.941279

Kim, S., Shin, Y., Kim, H., Pak, H., & Ha, J. (2013). Impacts of typhoon and heavy rain disasters on mortality and infectious diarrhea hospitalization in South Korea. *International Journal of Environmental Health Research*, *23*(5), 365–376. https://doi.org/10.1080/09603123.2012.733940

Kleinau, E., Post, M., & Rosensweig, F. (2004). Advancing Hygiene Improvement for diarrhea Prevention : Lessons learned. In *IRC International Water and Sanitation Centre*. Environmental Health Project (EHP). https://www.ircwash.org/resources/advancing-hygiene-improvement-diarrhea-prevention-lessons-learned

Kouadio, I. K., Aljunid, S. M., Kamigaki, T., Hammad, K., & Oshitani, H. (2012). Infectious diseases following natural disasters: prevention and control measures. *Expert Review of Anti-infective Therapy*, *10*(1), 95–104. https://doi.org/10.1586/eri.11.155

Kousky, C. (2016). Impacts of natural disasters on children. *The Future of Children*, *26*(1), 73–92. https://doi.org/10.1353/foc.2016.0004

Kraay, A. N. M., Man, O., Levy, M., Lévy, K., Ionides, E. L., & Eisenberg, J. N. S. (2020). Understanding the impact of rainfall on diarrhea: Testing the Concentration-Dilution hypothesis using a systematic review and Meta-Analysis. *Environmental Health Perspectives*, *128*(12). https://doi.org/10.1289/ehp6181

Kubo, T., Chimed- Ochir, O., Cossa, M., Ussene, I., Toyokuni, Y., Yumiya, Y., Kayano, R., & Salio, F. (2022). First activation of the WHO emergency medical team minimum data set in the 2019 response to tropical cyclone IDAI in Mozambique. *Prehospital and Disaster Medicine*, *37*(6), 727–734. https://doi.org/10.1017/s1049023x22001406

Lee, L. E. (1993). Active Morbidity Surveillance after Hurricane Andrew—Florida, 1992. *JAMA*, *270*(5), 591. https://doi.org/10.1001/jama.1993.03510050057027

Lemonick, D. M. (2007). Epidemics after natural disasters. *The Pediatric Infectious Disease Journal*, *26*(6), 552. https://doi.org/10.1097/inf.0b013e318054e34c

Lequechane, J. D., Mahumane, A., Chale, F., Nhabomba, C., Salomão, C., Lameira, C., Chicumbe, S., & Baltazar, C. S. (2020). Mozambique's response to cyclone Idai: how collaboration and surveillance with water, sanitation and hygiene (WASH) interventions were used to control a cholera epidemic. *Infectious Diseases of Poverty*, *9*(1). https://doi.org/10.1186/s40249-020-00692-5

Lévy, K., Woster, A. P., Goldstein, R. S., & Carlton, E. J. (2016). Untangling the Impacts of Climate Change on Waterborne Diseases: a Systematic Review of Relationships between Diarrheal Diseases and Temperature, Rainfall, Flooding, and Drought. *Environmental Science & Technology*, *50*(10), 4905–4922. https://doi.org/10.1021/acs.est.5b06186

Lin, J., King, R., Bhalla, N., & Brander, C. (2011). Assessment of prehospital care and disaster preparedness in a rural Guatemala clinic. *Prehospital and Disaster Medicine*, *26*(1), 27–32. https://doi.org/10.1017/s1049023x10000063

Loebach, P., & Korinek, K. (2019). Disaster vulnerability, displacement, and infectious disease: Nicaragua and Hurricane Mitch. *Population and Environment*, *40*(4), 434–455. https://doi.org/10.1007/s11111-019-00319-4

Lynch, V. D., & Shaman, J. (2023). Waterborne Infectious Diseases Associated with Exposure to Tropical Cyclonic Storms, United States, 1996–2018. *Emerging Infectious Diseases*, *29*(8). https://doi.org/10.3201/eid2908.221906

Ma, W., & Jiang, B. (2019). Health impacts due to major climate and weather extremes. In *Springer eBooks* (pp. 59–73). https://doi.org/10.1007/978-981-13-2583-0_4

Matsuda, I. (1993). Loss of human lives induced by the Cyclone of 29–30 April, 1991 in Bangladesh. *GeoJournal*, *31*(4), 319–325. https://doi.org/10.1007/bf00812781

Moll, D. M., Jacobson, J., McElroy, R. H., Sabogal, R., Finan, D. A., Haddock, D., Landry, C. C., & Perkins, M. (2001). Evaluation Of The Health Impact Of American Red Cross Posthurricane Mitch Water And Sanitation Reconstruction Efforts In Central America. *Proceedings of the Water Environment Federation*, *2001*(6), 436–459. https://doi.org/10.2175/193864701784291767

Moll, D. M., McElroy, R. H., Sabogal, R., Corrales, L. F., & Gelting, R. J. (2006). Health impact of water and sanitation infrastructure reconstruction programmes in eight Central American communities affected by Hurricane Mitch. *Journal of Water and Health*, *5*(1), 51–65. https://doi.org/10.2166/wh.2006.047

Morantz, C. (2005). CDC reports on illnesses in Hurricane Katrina evacuees and relief workers. *American Family Physician*, *72*(10), 2132. https://www.aafp.org/afp/2005/1115/p2132.html

Munnaf, M. F. (1998). Installation of simple water treatment unit at Cyclone-Aila affected Koyra region and assessment of DALYs lost due to diarrhoea [MA thesis, Khulna University of Engineering & Technology]. http://dspace.kuet.ac.bd/bitstream/handle/20.500.12228/71/Full%20Thesis.pdf?sequence=1

Murthy, S., & Christian, M. D. (2010). Infectious diseases following disasters. *Disaster Medicine and Public Health Preparedness*, *4*(3), 232–238. https://doi.org/10.1001/dmp.2010.hcn10005

Myint, N. W., Kaewkungwal, J., Singhasivanon, P., Chaisiri, K., Panjapiyakul, P., Siriwan, P., Mallik, A. K., Nyein, S. L., & Mu, T. T. (2011). Are there any changes in burden and management of communicable diseases in areas affected by Cyclone Nargis? *Conflict and Health*, *5*(1). https://doi.org/10.1186/1752-1505-5-9

Na, W., Lee, K. E., Myung, H., Jo, S., & Jang, J. (2017). Incidences of waterborne and foodborne diseases after meteorologic disasters in South Korea. *Annals of Global Health*, *82*(5), 848. https://doi.org/10.1016/j.aogh.2016.10.007

CDC (2005). Norovirus outbreak among evacuees from hurricane Katrina--Houston, Texas, September 2005. (2005). *Morbidity and Mortality Weekly Report (MMWR), 54(40), 1016–1018. Retrieved from https://www.cdc.gov/mmwr/preview/mmwrhtml/mm5440a4.htm*

Panda, S., Pati, K., Bhattacharya, M., Koley, H., Pahari, S., & Nair, G. B. (2011). Rapid situation & response assessment of diarrhoea outbreak in a coastal district following tropical cyclone AILA in India. *Indian Journal of Medical Research*, *133*(3), 395–400.

Partridge, R., King, K. M., & Proano, L. (2006). Medical support for emergency relief workers after typhoon Sudal in yap, micronesia. *Prehospital and Disaster Medicine*, *21*(3), 215–219. https://doi.org/10.1017/s1049023x00003708

Paul, B. K., Rahman, M., & Rakshit, B. C. (2010). Post-Cyclone Sidr illness patterns in coastal Bangladesh: an empirical study. *Natural Hazards*, *56*(3), 841–852. https://doi.org/10.1007/s11069-010-9595-5

Quinn, B. J., Baker, R., & Pratt, J. L. (1994). Hurricane Andrew and a pediatric emergency department. *Annals of Emergency Medicine*, *23*(4), 737–741. https://doi.org/10.1016/s0196-0644(94)70308-6

Quist, A., Fliss, M. D., Wade, T. J., Delamater, P. L., Richardson, D. B., & Engel, L. S. (2022). Hurricane flooding and acute gastrointestinal illness in North Carolina. *Science of the Total Environment*, *809*, 151108. https://doi.org/10.1016/j.scitotenv.2021.151108

Rabbaani, G., Huq, S., & Rahman, S. H. (1993). Impacts of climate change on water resources and human health: empirical evidences from a coastal district (Satkhira) in Bangladesh. In *Impact of Climate Change on Water and Health* (pp. 272–285). https://www.icccad.net/wp-content/uploads/2015/12/Impact-of-Climate-Ch-15-Saleemul-Huq-Article-Paper.pdf

Rafa, N., Jubayer, A., & Uddin, S. M. N. (2021). Impact of cyclone Amphan on the water, sanitation, hygiene, and health (WASH2) facilities of coastal Bangladesh. *Journal of Water Sanitation and Hygiene for Development*, *11*(2), 304–313. https://doi.org/10.2166/washdev.2021.170

Ramesh, B., Jagger, M. A., Zaitchik, B. F., Kolivras, K. N., Swarup, S., Deanes, L., & Gohlke, J. M. (2021). Emergency department visits associated with satellite observed flooding during and following Hurricane

Harvey. *Journal of Exposure Science and Environmental Epidemiology*, *31*(5), 832–841. https://doi.org/10.1038/s41370-021-00361-1

Ramesh, B., Jagger, M. A., Zaitchik, B. F., Kolivras, K. N., Swarup, S., Yang, B., Corpuz, B. G., & Gohlke, J. M. (2022). Estimating changes in emergency department visits associated with floods caused by Tropical Storm Imelda using satellite observations and syndromic surveillance. *Health & Place*, *74*, 102757. https://doi.org/10.1016/j.healthplace.2022.102757

Rath, B., Donato, J., Duggan, A., Perrin, K., Bronfin, D. R., Ratard, R., Vandyke, R., & Magnus, M. (2007). Adverse Health Outcomes after Hurricane Katrina among Children and Adolescents with Chronic Conditions. *Journal of Health Care for the Poor and Underserved*, *18*(2), 405–417. https://doi.org/10.1353/hpu.2007.0043

Ridpath, A., Bregman, B., Jones, L., Reddy, V., Waechter, H., & Balter, S. (2015). Challenges to Implementing Communicable Disease Surveillance in New York City Evacuation Shelters after Hurricane Sandy, November 2012. *Public Health Reports*, *130*(1), 48–53. https://doi.org/10.1177/003335491513000106

Salazar, M. A., Law, R., Pesigan, A., & Winkler, V. (2017). Health consequences of typhoon Haiyan in the eastern Visayas region using a syndromic surveillance. database. *PLOS Currents: Disasters,* 9. https://doi.org/10.1371/currents.dis.4a3d3b4474847b2599aa5c5eefe3a621

Sato, M., Nakamura, Y., Atogami, F., Horiguchi, R., Tamaki, R., Yoshizawa, T., & Oshitani, H. (2016). Immediate Needs and Concerns among Pregnant Women During and after Typhoon Haiyan (Yolanda). *PLOS Currents*. https://doi.org/10.1371/currents.dis.29e4c0c810db47d7fd8d0d1fb782892c

Saulnier, D. D., Ribacke, K. B., & Von Schreeb, J. (2017). No Calm After the Storm: A Systematic Review of Human Health Following flood and storm Disasters. *Prehospital and Disaster Medicine*, *32*(5), 568–579. https://doi.org/10.1017/s1049023x17006574

Savage, E., Christian, M. D., Smith, S., & Pannell, D. (2015). The Canadian Armed Forces medical response to Typhoon Haiyan. *Canadian Journal of Surgery*, *58*(3), S146–S152. https://doi.org/10.1503/cjs.013514

Setzer, C. N., & Domino, M. E. (2004). Medicaid Outpatient Utilization for Waterborne Pathogenic Illness following Hurricane Floyd. *Public Health Reports*, *119*(5), 472–478. https://doi.org/10.1016/j.phr.2004.07.004

Sheppa, C., Stevens, J., Philbrick, J. T., & Canada, M. (1993). The effect of a class IV hurricane on emergency department operations. *American Journal of Emergency Medicine*, *11*(5), 464–467. https://doi.org/10.1016/0735-6757(93)90084-o

Siddique, A. K., Islam, Q. R., Akram, K., Mazumder, Y., Mitra, A., & Eusof, A. (1989). Cholera epidemic and natural disasters; where is the link. *Tropical and Geographical Medicine, 41*(4), 377–382.

Simeon, D., Grantham- McGregor, S., Walker, S., & Powell, C. (1993). Effects of a hurricane on growth and morbidity in children from low-income families in Kingston, Jamaica. *Transactions of the Royal Society of Tropical Medicine and Hygiene*, *87*(5), 526–528. https://doi.org/10.1016/0035-9203(93)90073-y

Talukder, B., Van Loon, G. W., Hipel, K. W., Chiotha, S., & Orbinski, J. (2021). Health impacts of climate change on smallholder farmers. *One Health*, *13*, 100258. https://doi.org/10.1016/j.onehlt.2021.100258

Tyebally, A. (2009). *Cyclone Nargis—The Team Singapore Experience: Vol. (S1):s150-s151* (24th ed.). Cambridge University Press. https://doi.org/10.1017/S1049023X00056946

Van Loenhout, J. a. F., Cuesta, J. G., Abello, J., Isiderio, J. M., De Lara-Banquesio, M. L., & Guha-Sapir, D. (2018). The impact of Typhoon Haiyan on admissions in two hospitals in Eastern Visayas, Philippines. *PLOS ONE*, *13*(1), e0191516. https://doi.org/10.1371/journal.pone.0191516

Varo, R., Rodó, X., & Bassat, Q. (2019). Climate change, cyclones and cholera - Implications for travel medicine and infectious diseases. *Travel Medicine and Infectious Disease*, *29*, 6–7. https://doi.org/10.1016/j.tmaid.2019.04.007

Velimirović, B., & Mahadevan, S. (1972). The pattern of morbidity after typhoons in a tropical country. *International Journal of Biometeorology*, *16*(4), 343–360. https://doi.org/10.1007/bf01553619

Ventura, R. J. C., Muhi, E., De Los Reyes, V. C., Sucaldito, M., & Tayag, E. (2015). A community-based gastroenteritis outbreak after Typhoon Haiyan, Leyte, Philippines, 2013. *Western Pacific Surveillance and Response*, *6*(1), 1–6. https://doi.org/10.5365/wpsar.2014.5.1.010

Wang, W., Xun, H. M., Zhou, M., Jiang, B., Wang, S. W., Guo, Q., Kang, R. H., Wang, X., Marley, G., & Ma, W. (2015). Impacts of typhoon "Koppu" on infectious diarrhea in Guangdong province, China. *Biomedical and Environmental Sciences*, *28*(12), 920–923. *https://doi.org/10.3967/bes2015.127*

Yee, E., Palacio, H., Atmar, R. L., Shah, U. A., Kilborn, C., Faul, M. M., Gavagan, T., Feigin, R. D., Versalovic, J., Neill, F. H., Panlilio, A. L., Miller, M. D., Spahr, J., & Glass, R. I. (2007). Widespread Outbreak of Norovirus Gastroenteritis among Evacuees of Hurricane Katrina Residing in a Large "Megashelter" in

Houston, Texas: Lessons Learned for Prevention. *Clinical Infectious Diseases*, *44*(8), 1032–1039. https://doi.org/10.1086/512195

Zheng, J., Han, W., Jiang, B., Ma, W., & Zhang, Y. (2017). Infectious diseases and tropical cyclones in southeast China. *International Journal of Environmental Research and Public Health*, *14*(5), 494. https://doi.org/10.3390/ijerph14050494
